# Valorization of *Pimenta racemosa* Essential Oils and Extracts: GC-MS and LC-MS Phytochemical Profiling and Evaluation of *Helicobacter pylori* Inhibitory Activity

**DOI:** 10.3390/molecules27227965

**Published:** 2022-11-17

**Authors:** Iriny M. Ayoub, Marwa M. Abdel-Aziz, Sameh S. Elhady, Alaa A. Bagalagel, Rania T. Malatani, Wafaa M. Elkady

**Affiliations:** 1 Department of Pharmacognosy, Faculty of Pharmacy, Ain Shams University, Cairo 11566, Egypt; 2 Regional Center for Mycology and Biotechnology (RCMB), Al-Azhar University, Cairo 11651, Egypt; 3 Department of Natural Products, Faculty of Pharmacy, King Abdulaziz University, Jeddah 21589, Saudi Arabia; 4 Department of Pharmacy Practice, Faculty of Pharmacy, King Abdulaziz University, Jeddah 21589, Saudi Arabia; 5 Department of Pharmacognosy and Medicinal Plants, Faculty of Pharmacy, Future University in Egypt, Cairo 11835, Egypt

**Keywords:** *Pimenta racemosa*, LC-MS, GC-MS, anti-*Helicobacter pylori*, molecular modeling, drug discovery, health care

## Abstract

*Pimenta racemosa* is a commonly known spice used in traditional medicine to treat several ailments. In this study, comprehensive phytochemical profiling of the essential oils and methanol extracts of *P. racemosa* leaves and stems was performed, alongside assessing their potential *Helicobacter pylori* inhibitory activity in vitro and in silico. The essential oils were chemically profiled via GC-MS. Moreover, the methanol extracts were profiled using HPLC-PDA-ESI-MS/MS. The antibacterial activity of the essential oils and methanol extracts against *H. pylori* was determined by adopting the micro-well dilution method. GC-MS analysis unveiled the presence of 21 constituents, where eugenol represented the major component (57.84%) and (59.76%) in both leaves and stems of essential oils, respectively. A total of 61 compounds were annotated in both leaves and stems of *P. racemosa* methanolic extracts displaying richness in phenolic compounds identified as (*epi*)catechin and (*epi*)gallocatechin monomers and proanthocyanidins, hydrolyzable tannin derivatives (gallotannins), flavonoids, and phenolic acids. The stem essential oil showed the most promising inhibitory effects on *H. pylori,* exhibiting an MIC value of 3.9 µg/mL, comparable to clarithromycin with an MIC value of 1.95 µg/mL. Additionally, in silico molecular modeling studies revealed that decanal, eugenol, terpineol, delta-cadinene, and amyl vinyl showed potential inhibitory activity on *H. pylori* urease as demonstrated by high-fitting scores indicating good binding to the active sites. These findings indicate that *P. racemosa* comprises valuable phytochemical constituents with promising therapeutic effects, particularly the stem, an economic agro-industrial waste.

## 1. Introduction

*Pimenta racemosa* (Mills) J.W. Moore, a member of the family Myrtaceae, is one of the most commonly known spices. It is also called the Bay or Lemon Bay Rum Spice tree [1]. It is up to 25 ft high. This species is native to the Caribbean region but can be adopted in different warm climates [2]. Traditionally in Egypt and other countries as Cuba, Bahamas and Jamaica, *P. racemosa* leaves have been prepared as tea and used to treat flatulence, colds, or fever [3]. This activity could be assigned to the richness of essential oils and phenolic constituents [4]. The anti-inflammatory and antinociceptive activities of the aqueous leaves extract were also reported [3]. Furthermore, *P. racemosa* exhibited antimicrobial, antioxidant, analgesic, and anti-inflammatory properties [5]. Polyphenolics have been generally recognized for their beneficial effects on several health problems, including diabetes, cardiovascular diseases, neurodegenerative diseases, cancers, and osteoporosis [6]. This is usually due to their free radical scavenging activity, reducing the risk of different ailments [7]. In addition, essential oils have long been known for their therapeutic activities and their use in the food and cosmetics industries [5].

*Helicobacter pylori* is a gram-negative bacteria with a spiral shape responsible for several illnesses, including asymptomatic gastritis, chronic inflammation, and gastric cancer [8]. Despite the improvements in antimicrobial treatments worldwide, there is still no model therapy for *H. pylori* infection due to drug resistance and numerous side effects of the antibiotics [9]. Plants have usually been considered alternative or complementary medicine for many ailments. Research has been conducted to find a suitable cure for *H. pylori* from natural sources. The research literature revealed that *P. racemosa* leaves and fruits exhibited promising anti-inflammatory and antimicrobial activities [3,5,10]. Moreover, *P. racemosa* bark also showed strong anti-schistosomal activity [11].

Leaves and fruits are the most widely used parts of *P. racemosa* [1]. The stem could be considered a natural and economic agro-industrial waste. However, it could play a role in phytotherapy and the pharmaceutical industry. Hence, the present study aimed to compare the phytochemical profile of the methanol extracts and essential oils of both leaves and stems. Moreover, the potential anti-*H. pylori* inhibitory activity was assessed in vitro, alongside the evaluation of the in silico inhibitory activity of the identified phytoconstituents.

## 2. Results and Discussion

### 2.1. GC/MS Analysis of the Essential Oils

**Figure 1:**
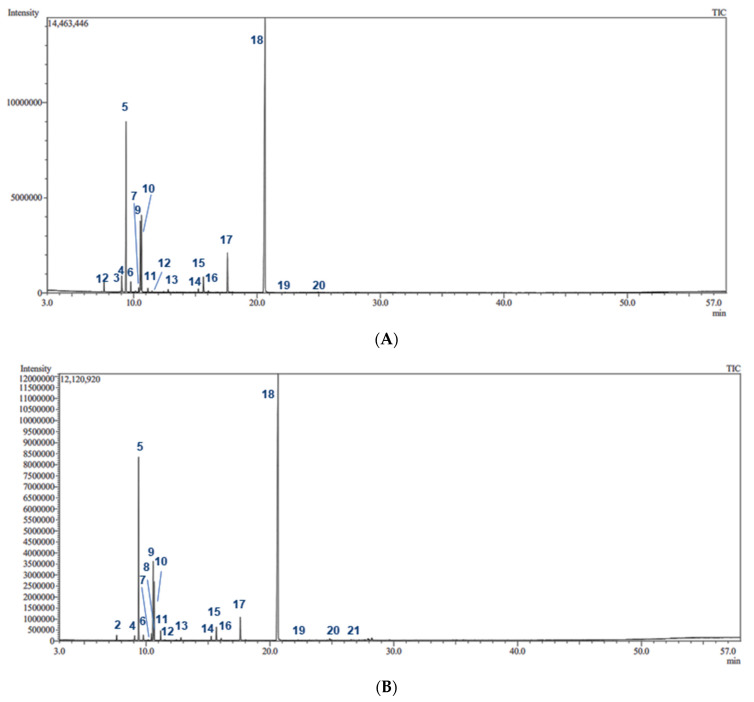
GC-MS chromatograms of *P. racemosa* leaf (**A**) and stem (**B**) essential oils on the Rtx-5MS column.

**Figure 2:**
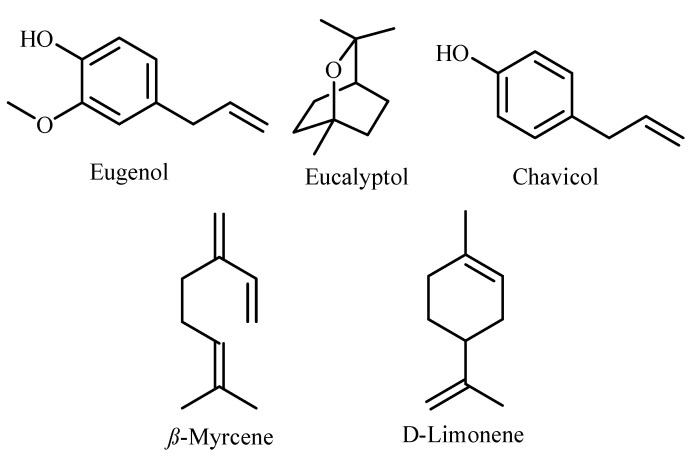
Major constituents identified in *P. racemosa* leaf and stem essential oils.

Hydrodistillation of fresh *P. racemosa* leaves and stems yielded 0.61% and 0.06% (*w*/*w*) essential oil, respectively. Essential oils were pale yellow with a distinctive strong aromatic clove-like odor. GC/MS analysis of the essential oils identified 21 volatile components in the leaves, accounting for 99.57% of the oil composition. Meanwhile, 19 compounds were identified in *P. racemosa* stem essential oil, representing 98.82% of the total oil composition (Table 1, Figure 1 and Figure 2).

Phenyl propanoids represented the most abundant class identified herein. Eugenol was the main compound recognized in both leaf and stem essential oils representing 57.84% and 59.76% of the total oil composition, respectively. Chavicol, a key marker for *P. racemosa* essential oil [1], was detected in both leaves (4.18%) and stems’ (2.6%) essential oils. Oxygenated monoterpene hydrocarbons could be detected in both leaves (9.33%) and stems (7.37%), represented by eucalyptol, *α*-terpineol, and terpinen-4-ol. Monoterpene hydrocarbons were also found in a considerable amount, representing 26.48% in leaves and 28.04% in stems, rich in *β*-Myrcene and D-limonene. However, sesquiterpene hydrocarbons were presented in trace amounts. The major components identified are represented in Figure 2.

Our results complied with earlier reports on the chemical composition of *P. racemosa* growing in different regions. Earlier reports showed the effect of the geographical source on essential oil composition, where *P. racemosa* leaves from two different locations in Benin yielded a range from 0.9 to 2.4% (*w*/*w*) [12], whereas that from North India yielded 0.02% (*w*/*w*) [13]. Besides the essential oils from *P. racemosa* leaves grown in Venezuela, different locations in Benin, India, and Egypt showed richness in eugenol content with variable percentages [1,12,13,14].

### 2.2. HPLC-PDA-ESI-MS/MS Analysis

**Figure 3:**
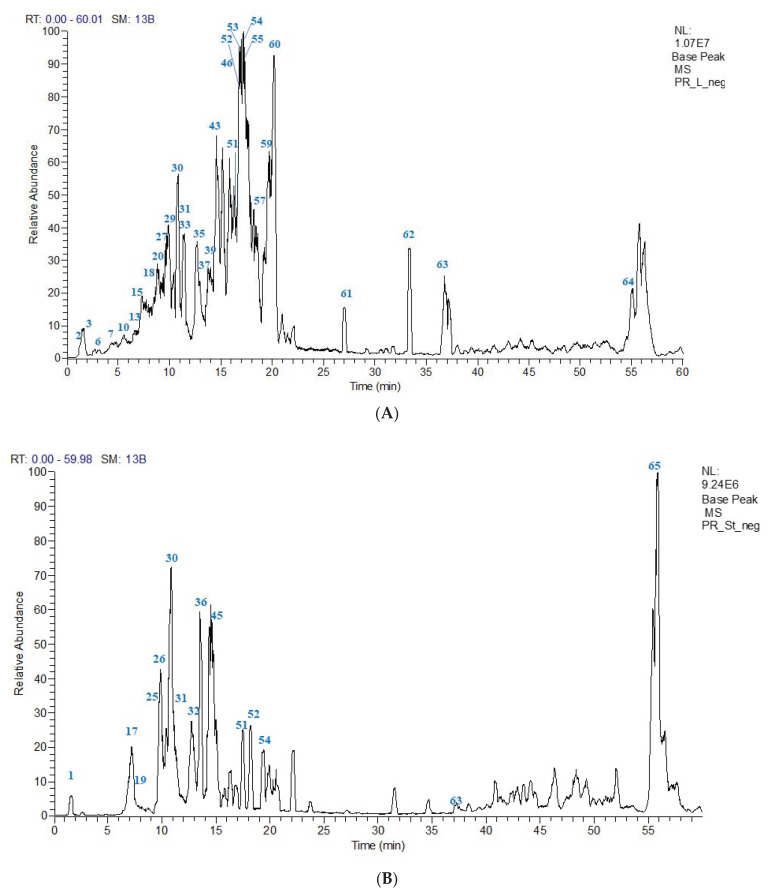
(**A**) UPLC-ESI-MS base peak chromatogram of *P. racemosa* leaves methanol extract (PRL-Me) in the negative ion mode. (**B**) UPLC-ESI-MS base peak chromatogram of *P. racemosa* stem methanol extract (PRS-ME) in the negative ion mode. Peaks are numbered relative to compounds listed in Table 2.

**Figure 4:**
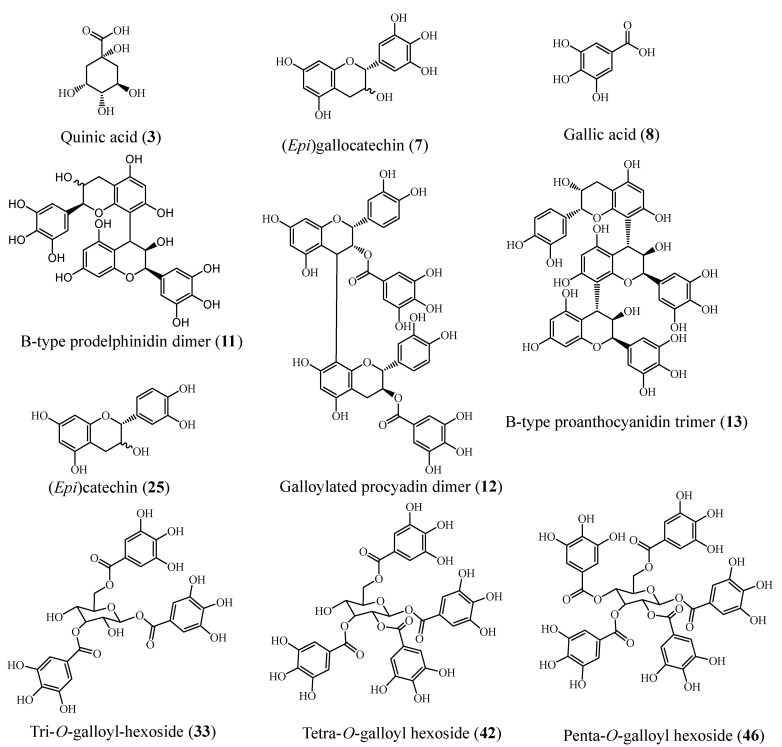
Representative compounds identified in *P. racemosa* leaf and stem methanol extracts.

Metabolic profiling of both leaves and stems of *P. racemosa* methanolic extracts was achieved using HPLC-PDA-ESI-MS/MS. Sixty-one compounds were annotated. Most of them belonged to the class of polyphenolics, including (epi)catechin and (epi)gallocatechin monomers and proanthocyanidins (the oligomeric polyflavan-3-ol). Moreover, hydrolyzable tannin derivatives (gallotannins), phenolic acids & flavonoids were also annotated (Table 2 and Figure 3). Compounds were tentatively identified based on the mass of the molecular ion peaks, their tandem mass data, considering fragmentation patterns, neutral mass losses, UV spectra, and comparison with bibliographic references [15,16,17,18,19]. Chemical structures of representative compounds identified in *P. racemosa* leaf and stem methanol extracts are displayed in Figure 4.

#### 2.2.1. Proanthocyanidins

Several classes of proanthocyanidins (PAs) could be detected herein. Proanthocyanidins constituted of (epi)catechin units are known as procyanidins, while proanthocyanidins composed of (epi)gallocatechin units were designated as prodelphinidins. HPLC-PDA-ESI-MS/MS analysis showed a series of derivatives of polyflavan-3-ol. The pseudomolecular ion [M−H]^−^ at *m*/*z* 289 was tentatively identified as (epi)catechin, while [M−H]^−^ ion at *m*/*z* 305 was annotated as (epi)gallocatechin. Proanthocyanidins, including dimers, trimers, tetramers, and pentamers, alongside different galloylated procyanidins and prodelphinidins, were annotated in *P. racemosa* leaves and stem extracts.

#### 2.2.2. Organic Acids, Phenolic Acids and Their Derivatives

Herein, several organic acids and phenolic acids were annotated. Among them, compound **3** showed a pseudomolecular ion [M−H]^−^ at *m*/*z* 191 corresponding to a deprotonated quinic acid [quinic acid−H]^−^ and a product ion at *m*/*z* 173 representing [quinic acid−H−H_2_O]^−^ ion was observed [20]. Compound **8** showed a parent ion at *m*/*z* 169, typical for gallic acid compared to its mass spectra in literature [10]. Compounds **51** and **56** were annotated as gallic acid dihexoside isomers exhibiting a base peak of the deprotonated molecule at *m*/*z* 493 and a product ion at *m*/*z* 169 of deprotonated gallic acid resulting from the loss of the two hexose units [20].

Moreover, six gallotannins were identified; tri-O-galloyl-hexoside (**33** and **38**) showed pseudomolecular ion at *m*/*z* 635; and fragment ions at *m*/*z* 483 and 465, corresponding to the loss of a galloyl (digalloylglucose product ion) and a gallate unit respectively. The same principle was applied to recognize pseudomolecular ions *m*/*z* 787, 939 corresponding to tetra-O-galloyl-hexoside (**42** and **44**) and penta-O-galloyl-hexoside (**46** and **50**), respectively.

#### 2.2.3. Flavonoids

Quercetin glycosides were the main identified flavonol glycosides. Compound **45** was identified as quercetin-O-hexoside displaying a pseudomolecular ion at *m*/*z* 463. Compound **54** was annotated as quercetin-O-pentoside displaying a pseudomolecular ion at *m*/*z* 433 and quercetin-O-deoxyhexoside (**55**) at *m*/*z* 447. All revealed quercetin aglycone displaying a characteristic product ion at *m*/*z* 301. The sugar moieties could be annotated by calculating the losses of the sugar part, that is, 162 amu (hexose), 152 amu (pentose), and 146 amu (deoxyhexose) [21]. Additionally, compound (**57**) exhibited a pesudomolecular ion peak at *m*/*z* 625 and a base peak at *m*/*z* 463, inferring the loss of one hexose unit, and a product ion at *m*/*z* 301, implying the loss of a second hexose and denoting quercetin aglycone [M−H−162−162]^−^. Thus, compound **57** was tentatively identified as quercetin-di-O-hexoside [22].

**Table 2:** Identified metabolites in *P. racemosa* leaf methanol extract (PRL-ME) and stem methanol extract (PRS-ME) using HPLC-PDA-ESI-MS/MS in negative ion mode.

	Compound	Rt (min)	UV λ (nm)	[M−H]^−^	(PRL-ME)	(PRS-ME)	Fragment Ions (MS/MS)	Class	Ref.
1.	B-type proanthocyanidin pentamer	1.37	279	1425	-	+	1257, 1187, 1155	Proanthocyanidin	[23]
2.	Caffeoylglucaric acid	1.42	236, 270	371	+	-	325, 191	Phenolic acid	
3.	Quinic acid	1.47	236, 265	191	+	-	173	Organic acid	[20]
4.	B-type proanthocyanidin trimer (EC→EG→EG)	1.52	267	897	+	-	879, 711, 693, 543, 407, 289	Proanthocyanidin	
5.	B-type proanthocyanidin dimer (EC→EG)	1.61	275	593	+	-	575, 467, 441, 305, 289	Proanthocyanidin	
6.	Galloylated prodelphinidin dimer (EG→EG)2 g	4.17	273	914	+	-	727, 559, 423, 305	Proanthocyanidin	[23]
7.	(*Epi*)gallocatechin	4.28	273	305	+	-	287, 261, 221, 219, 179, 165, 125	Flavonoid	[23,24]
8.	Gallic acid	4.68	270	169	+	-	125	Phenolic acid	[25]
9.	(*Epi*)gallocatechin	5.46	273	305	+	-	287, 261, 221, 219, 179, 165, 125	Flavonoid	
10.	B-type Procyanidin dimer (EC→EC)	5.71	274	577	+	+	559, 451, 425, 407, 299, 289, 287	Proanthocyanidin	[23,26]
11.	B-type Prodelphinidin dimer (EG→EG)	5.96	274	609	+	-	591, 483, 441, 423, 305	Proanthocyanidin	[23]
12.	Galloylated procyanidin dimer (EC→EC)2 g	6.22	276	881	+	-	729, 711	Proanthocyanidin	[27]
13.	B-type proanthocyanidin trimer EG→EG→EC	6.63	277	897	+	+	879, 771, 729, 711, 593, 407, 289	Proanthocyanidin	[27,28]
14.	B-type procyanidin trimer (EC→EC→EC)	6.83	278	865	+	+	847, 695, 577, 449, 407, 287	Proanthocyanidin	[29]
15.	Prodelphinidin trimer (EG→EG→EG)	7.04	277	913	+	-	895, 787, 745, 727, 609, 559, 483, 305	Proanthocyanidin	[23]
16.	B-type procyanidin trimer (EC→EC→EC)	7.09	277	865	+	-	847, 695, 577, 407, 287	Proanthocyanidin	
17.	A-type procyanidin trimer EC→EC→EC	7.11	277	863	-	+	737, 711, 693, 591, 575, 289	Proanthocyanidin	[29]
18.	B-type Procyanidin dimer (EC→EC)	7.14	277	577	+	-	559, 451, 425, 407, 299, 289, 287	Proanthocyanidin	
19.	Proanthocyanidin dimer EC→EG	7.76	278	593	+	+	575, 467, 425, 407, 305, 289, 245	Proanthocyanidin	[27]
20.	(*Epi*)gallocatechin	8.2	278	305	+	-	287, 261, 221, 219, 179	Flavonoid	[23,24]
21.	B-type proanthocyanidin trimer EG→EG→EC	8.3	277	897	+	-	879, 771, 729, 711, 593, 577, 305, 289	Proanthocyanidin	[27]
22.	B-type Procyanidin dimer (EC→EC)	8.55	277	577	+	-	559, 451, 425, 407, 299, 289, 287	Proanthocyanidin	
23.	B-type proanthocyanidin dimer (EC→EG)	8.79	278	593			575, 467, 441, 407, 305, 289	Proanthocyanidin	
24.	B-type proanthocyanidin trimer EC→EC→EG	9.54	278	881	+	-	755, 729, 711, 695, 593, 425, 407, 289	Proanthocyanidin	[23]
25.	(*Epi*)catechin	9.73	278	289	+	+	245, 205, 179	Flavonoid	[24,29]
26.	B-type procyanidin tetramer EC→EC→EC→EC	9.81	278	577	-	+	559, 451, 425, 407, 299, 289, 287	Proanthocyanidin	[23]
27.	B-type Procyanidin dimer (EC→EC)	9.9	278	577	+	-	559, 451, 425, 407, 299, 289, 287	Proanthocyanidin	[23]
28.	B-type procyanidin tetramer EC→EC→EC→EC	10.21	278	1153	+	+	983, 863,695, 575	Proanthocyanidin	[23]
29.	B-type proanthocyanidin dimer (EC→EG)	10.26	278	593	+	+	575, 467, 441, 407, 305, 289	Proanthocyanidin	[27]
30.	(*Epi*)catechin	10.82	278	289	+	+	245, 205, 179, 151	Flavonoid	[24,30]
31.	B-type procyanidin trimer EC→EC→EC	10.86	278	865	+	+	847, 695, 577, 575, 407, 289	Proanthocyanidin	[23]
32.	Galloylated procyanidin dimer (EC→EC)g	11.00	278	729	+	+	711, 603, 577, 559, 425, 407, 289	Proanthocyanidin	[26]
33.	Tri-*O*-galloyl-hexoside	11.1	278	635	+	-	483, 465	Gallotannin	[20]
34.	Galloylated procyanidin trimer (EC→EC→EC)→2 g	11.15	278	1169	+	-	1042,890, 864, 703, 633, 443, 424	Proanthocyanidin	[23]
35.	B-type proanthocyanidin dimer EA→EC	11.31	278	559	+	-	541, 453, 407, 321, 289	Proanthocyanidin	
36.	B-type procyanidin pentamer EC→EC→EC→EC→EC	11.49	278	1441	-	+	1421, 1315, 1271, 1153, 1151, 1027, 865, 863,739, 575	Proanthocyanidin	[23]
37.	Galloylated procyanidin trimer (EC→EC→EC)g	11.75	277	1017	+	-	999, 891, 865, 739, 729, 575, 425, 407	Proanthocyanidin	[23]
38.	Tri-*O*-galloyl-hexoside isomer	12.38	278	635	+	-	483, 465	Gallotannin	
39.	B-type Procyanidin dimer (EC→EC)	12.43	278	577	+	-	559, 451, 425, 407, 299, 289, 287	Proanthocyanidin	[23]
40.	Galloylated procyanidin dimer (EC→EC)g	12.66	277	729	+	+	711, 603, 577, 559, 425, 407, 289	Proanthocyanidin	[23]
41.	B-type Procyanidin dimer (EC→EC)	12.79	279	577	-	+	559, 451, 425, 407, 299, 289, 287	Proanthocyanidin	[23]
42.	Tetra-*O*-galloyl hexoside	13.12	277	787	+	-	635, 617, 465, 331, 313	Gallotannin	[20]
43.	Galloylated procyanidin trimer (EC→EC→EC)g	13.82	278	1017	+	+	999, 891, 865, 847, 739, 729, 695, 677, 575	Proanthocyanidin	[23]
44.	Tetra-*O*-galloyl hexoside isomer	14.57	274	787	+	-	635, 617, 465, 331, 313	Gallotannin	
45.	Quercetin-*O*-hexoside	14.59	274, 349	463	+	+	301, 179, 151	Flavonoid	[20]
46.	Penta-*O*-galloyl hexoside	14.80	274	939	+	-	921, 787, 769, 635, 617, 555, 465, 447, 313, 295	Gallotannin	[20]
47.	Quercetin-*O*-galloyl hexoside	15.15	272, 351	615	+	-	463, 301, 300, 179	Flavonoid	[31]
48.	Pentahydroxyflavone-*C*-hexoside	15.70	266, 353	463	+	-	445, 373, 343, 301, 179, 151, 133	Flavonoid	
49.	Pentahydroxyflavone-*C*-pentoside	15.83	271, 352	433	+	-	415, 373, 343, 301, 300, 287, 251, 193, 179, 151, 125	Flavonoid	
50.	Penta-*O*-galloyl hexoside	15.96	268, 353	939	+	-	921, 787, 769, 635, 617, 555, 465, 447, 313	Gallotannin	[20]
51.	Gallic acid dihexoside	16.11	266	493	+	+	341, 313, 179, 169	Phenolic acid	[20]
52.	Quercetin-*O*-deoxyhexoside	16.55	255, 353	447	+	+	301, 255, 179, 151	Flavonoid	[10,20]
53.	Ellagic acid-*O*-pentoside	16.88	266, 351	433	+	-	301, 191, 169	Phenolic acid	
54.	Quercetin-*O*-pentoside	16.92	268, 349	433	+	+	415, 301, 300, 179, 151	Flavonoid	
55.	Quercetin-*O*-deoxyhexoside	17.05	264, 348	447	+	-	301, 255, 179, 151	Flavonoid	
56.	Gallic acid dihexoside isomer	17.21	273	493			341, 313, 179, 169, 151	Phenolic acid	[20]
57.	Quercetin-di-*O*-hexoside	18.25	271, 350	625	+	-	463, 301, 179	Flavonoid	[22]
58.	Gallic acid derivative	19.23	268, 348	477	+	-	313, 301, 223, 169	Phenolic acid	[31,32,33]
59.	Gallic acid derivative	19.38	268, 336	447	+	-	313, 301, 269, 169, 125	Phenolic acid	
60.	Quercetin *O*-acetyl-deoxyhexoside	20.25	275, 350	489	+	-	471, 447, 301, 300, 179, 151	Flavonoid	
61.	Unidentified	27.03	275	313	+	-	313, 298, 283, 269, 257, 243, 227, 163, 135, 113		
62.	Unidentified	33.32	291, 311	289	+	-	245, 163, 119		
63.	Unidentified	36.67	279	325	+	+	325, 310, 307, 295, 281, 252, 191		
64.	Hydroxypalmitic acid	48.09		271	+	-	271, 253, 225	Fatty acid	
65.	Unidentified	55.83		817	-	+	796, 711		

### 2.3. Evaluation of Anti-H. pylori Activity

*P*. *racemosa* leaves and stems essential oils displayed higher *H. pylori* inhibitory activity than the corresponding methanol extracts (Table 3). Interestingly, the essential oil isolated from the stems elicited the highest activity exhibiting a MIC of 3.9 µg/mL comparable to clarithromycin (MIC 1.95 µg/mL).

The promising inhibitory activity of essential oils perceived herein could be ascribed to the high content of phenyl propanoids, monoterpenes, and oxygenated monoterpenes. Eugenol, the main identified compound in both essential oils, could be the reason for such a great activity. It can decrease the viability of *H. pylori*, regardless of the strain [34]. Furthermore, eugenol can generate morphological alterations in some enzymes in the cell wall because of hydrogen bond formation between the phenolic hydroxyl group and the enzyme [14].

Reports have stated that terpenes have a bactericidal effect; this could be due to their nature [35]. Their solubility in water is weak to moderate, but they are readily dissolved in the lipid layer of the biological membranes. This could affect the cell wall permeability, disrupting the lipid structure and inhibiting microbial metabolism. Additionally, monoterpenes have anti-ulcerogenic and healing effects [35]. The anti-*H. pylori* activity of the essential oils is usually correlated to certain terpenoid components such as α-pinene, β-pinene, and myrcene [8]. Myrcene is present in a considerable amount in both essential oils isolated from leaves and stems, 16.30 and 17.43%, respectively.

Moreover, polyphenolic compounds and tannin content in the methanol extracts of both leaves and stems could be accountable for the observed anti-*H. pylori* activity [9].

### 2.4. In Silico Evaluation of Anti-H. pylori Activity

**Figure 5:**
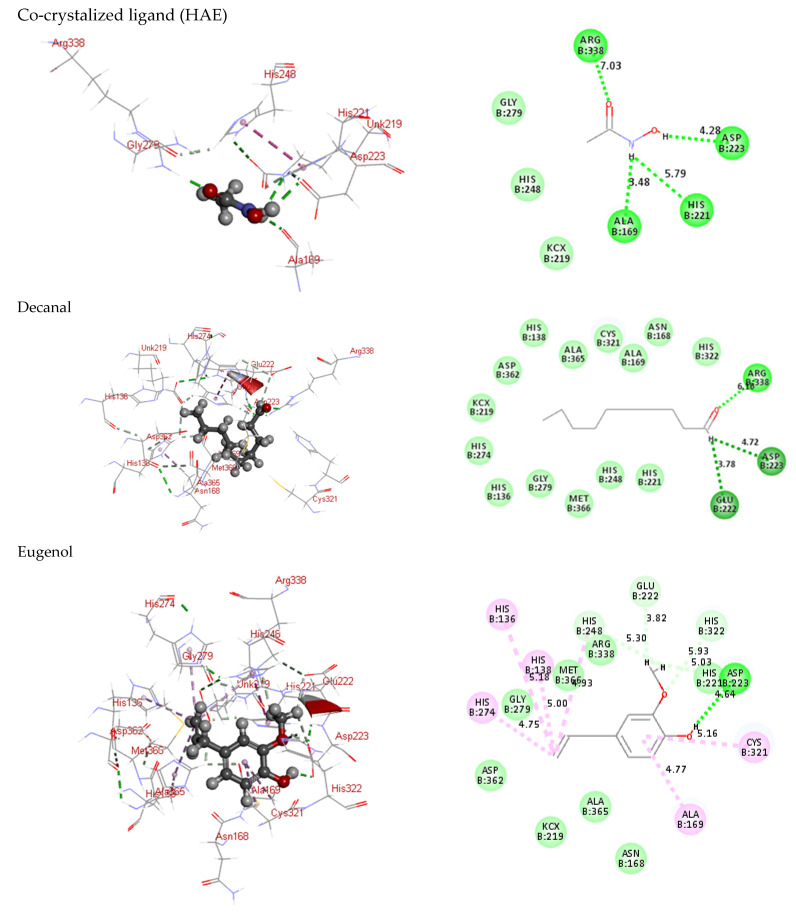

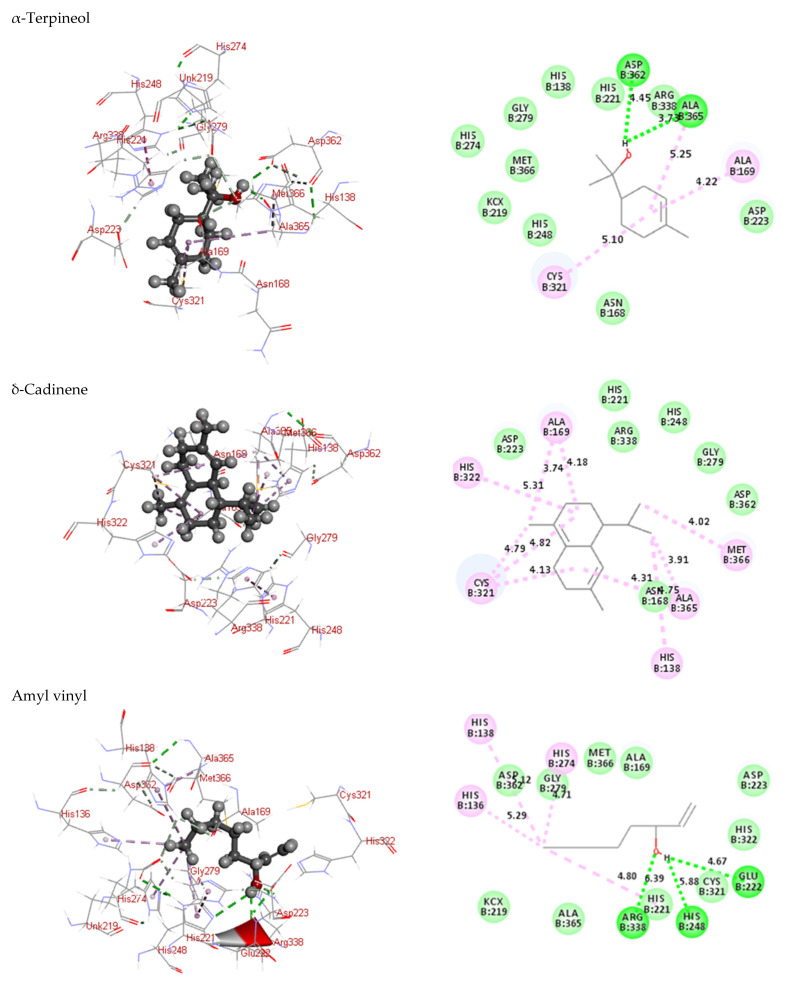
Two-dimensional and three-dimensional binding modes of identified compounds in *P. racemosa* leaf and stem essential oils within *H. pylori* urease (1E9Y) active site employing C-docker protocol.

*P. racemosa* essential oils exhibited notable *H. pylori* inhibitory activity; thus, an in-silico study was conducted to validate the obtained results. *H. pylori* urease crystal structure was obtained from the Protein Data Bank (http://www.rcsb.org/pdb/ accessed on 20 June 2022) provided with HAE (PDB ID 1E9Y; 3.00 Å). The acetohydroxamic acid (HAE), a co-crystallized ligand, was utilized to identify the amino acid residues constituting the urease active binding site. The 1E9Y protein was used in the docking research. The amino acid Arg338 was involved in creating bonds with the chemicals investigated. The computed free binding energies of phytoconstituents identified in *P. racemosa* essential oils ranged from −29.76 to −13.31 kcal/mol employing both pH-based and rule-based ionization modes (Table 4). The pH-based ionization mode mimics the physiological pH [36]. Meanwhile, the ionization of functional groups as well as amino acid moieties at the active site is explained well by the rule-based ionization method [37]. These values indicated that the identified phytochemicals bind well to the urease active site. Decanal, eugenol, terpineol, δ-cadinene, and amyl vinyl had the best affinity and orientation (Figure 5). These compounds were found in both essential oils displaying a higher percentage in the stem essential oil, representing 0.24, 59.76, 1.49, 0.10, and 0.45%, respectively, of the total oil composition. The occurrence of alcoholic, phenolic, and ketonic groups in the identified volatile oil components allows the formation of hydrogen and ionic bonds with various amino acids. These interactions can efficiently engage with the proteins’ binding sites, causing their 3D shape (conformation) to be disrupted, resulting in activity inhibition [38]. Consequently, it is possible to conclude that the phytoconstituents discovered herein can act as potential inhibitors of *H. pylori* urease.

## 3. Materials and Methods

### 3.1. Plant Material

*P. racemosa* leaves and stems were collected in June 2017 from Orman Botanical Garden in Giza, Egypt, and identified by Mrs. Trease Labib, a Plant Taxonomy Consultant at the Egyptian Ministry of Agriculture and Land Reclamation. A voucher specimen (PHG-P-PR-359) was deposited at the herbarium, Ain Shams University, Department of Pharmacognosy, Faculty of Pharmacy.

### 3.2. Essential Oils Isolation

Fresh *P. racemosa* leaves and stems were hydrodistilled for 4 h using the Clevenger apparatus to isolate their essential oils PRL-EO and PRS-EO, respectively. The yield (% *w*/*w*) per hundred grams of plant material was determined in triplicate. Both isolated oils PRL-EO and PRS-EO were dried over anhydrous Na_2_SO_4_ and preserved in tightly sealed amber glass vials for further analyses.

### 3.3. Preparation of Plant Extracts

Air-dried leaves (50 g) and stems (20 g) were ground and then extracted using methanol (500 mL × 3; 25–27 °C) for 48 h. Extracts were filtered, then concentrated under vacuum using a rotary evaporator (BUCHI Labortechnik, Flawil, Switzerland) at a temperature of 45 °C. Then, the dried extracts were lyophilized employing an Alpha 1-4 LSC Christ freeze dryer (Martin Christ Gefriertrocknungsanlagen GmbH, Osterode, Germany) to yield 4.8 g of *P. racemosa* leaf extract (PRL-ME) and 1.5 g of the stem extract (PRS-ME). Extraction was performed in triplicate. Extracts were preserved in tightly sealed containers at 4 °C until further analysis.

### 3.4. GC/MS Analysis of Essential Oils

A Shimadzu GCMS-QP 2010 (Kyoto, Japan) equipped with a Rtx-5MS capillary column (30 m length with 0.25 mm I.D. and 0.25 m film thickness; Restek, Bellefonte, PA, USA) was used to analyze essential oil samples PRL-EO and (PRS-EO). The oven temperature was set to 45 °C for 2 min, then increased to 300 °C at a rate of 5 °C/min and maintained at 300 °C for another 5 min; the injector temperature was set to 250 °C. Helium was used as a carrier gas at a flow rate of 1.41 mL/min. Diluted samples (1% *v*/*v*) were injected (1 μL) at a split ratio of 15:1. Mass spectra were acquired in the range of 35–500 amu, EI mode: 70 eV. The interface and the ion source temperatures were set to 280 °C and 200 °C, respectively. The essential oils were analyzed independently, and the reported data represented the average of the three readings.

### 3.5. Identification of Essential Oil Components

Peaks were first deconvoluted using AMDIS software, afreely available software on www.amdis.net (accessed on 20 June 2022). Identification of essential oil phytoconstituents was achieved by comparing their mass spectral profiles with mass spectra within the NIST-17 GC-MS database (NIST, Gaithersburg, MD, USA) and literature [39,40,41,42,43,44,45,46]. Retention indices (RI) were calculated from the retention times of C8-C28 all-even *n*-alkanes injected under the same conditions.

### 3.6. HPLC-PDA-ESI-MS/MS Analysis

*P. racemosa* leaves and stems (PRL-ME) and PRS-ME methanol extracts were analyzed by HPLC-PDA-ESI-MS/MS, as described by Elkady et al. [21]. Chromatographic separations, UV, and mass spectral analyses were achieved using a Finnigan Surveyor HPLC system composed of a MS pump plus, autosampler, and PDA detector plus equipped with an EC 150/3 Nucleodur 100-3 C18 column (Macherey-Nagel, Dueren, Germany) coupled to a Finnigan LCQ-Duo ion trap with an ESI source mass spectrometer (Thermo Quest, San Jose, CA, USA). Data acquisitions and analyses were performed using XcaliburTM ver. 2.0.7platform (Thermo Scientific, Waltham, MA, USA).

### 3.7. Evaluation of Anti-H. pylori Activity

Anti-*H. pylori* activity was determined using the MTT assay to assess the minimum inhibitory concentration (MIC) for bacterial growth: a series of concentrations with a final concentration range from 125 to 0.24 μg/mL was prepared for the methanol extracts, tested oils, or reference drug clarithromycin in dimethyl sulfoxide (DMSO).

The micro-well dilution method was used to assess the potential *H. pylori* (ATCC 43504) inhibitory activity of *P. racemosa* essential oils and methanol extracts PRL-EO, PRS-EO, PRL-ME, and PRS-ME [8]. A 10 µL inoculum of *H. pylori* (10^6^ CFU/mL) was added to 40 μL of the Brain Heart Infusion (BHI) growth medium in 10% fetal bovine serum (FBS) in each well. Subsequently, 50 μL aliquots of two-fold serial dilutions of test samples and Clarithromycin (standard reference) in dimethyl sulfoxide (125–0.24 µg/mL) were added. DMSO and Clarithromycin were used as negative and positive controls, respectively. The plates were incubated in an 85% N_2,_ 10% CO_2_, and 5% O_2_ atmosphere at 37 °C for 3 days. Afterward, 3-(4,5-dimethyl-thiazol-2-yl)-2,5-diphenyl- tetrazolium bromide (MTT) reagent at a freshly prepared 0.5 mg/mL concentration in water and 40 μL were added to each well, then incubated for 30 min where purple color indicated bacterial growth. Absorbance was recorded at 620 nm using an ELISA microplate reader.

The inhibition percentage was calculated from the equation:

% inhibition = [(Absorbance of Control − Absorbance of Sample)/Absorbance of Control] × 100.

The MIC was defined as the lowest concentration, where no color change of MTT (inhibitory percentages 100%) was observed [8].

### 3.8. In Silico Molecular Docking Study

The molecular binding mode of the major identified compounds in the essential oils of *P. racemosa* leaves and stems to the crystal structure of *H. pylori* urease was assessed for their putative *H. pylori* inhibitory activity using Discovery Studio 4.5 (Accelrys Software, Inc., San Diego, CA, USA). The crystal structure Urease X-ray (PDB ID: 1E9Y; 3.00Å) was obtained from the Protein Data Bank (http://www.rcsb.org/pdb/ accessed on 20 June 2022). Enzyme preparation was performed through the elimination of all water molecules. Hydrogen atoms were subsequently added, and then the protein structure was refined.

The target binding sites in urease were defined based on the interaction of acetohydroxamic acid (HAE), the co-crystallized inhibitor, and the enzyme. Prior to docking simulations, the co-crystallized ligand was removed. ChemDraw Ultra 8.0.3 was used to construct the 2D structures of the compounds identified in both essential oils. Subsequently, ligands were prepared adopting the ligand preparation protocol in Discovery Studio employing rule-based and pH-based ionization methods. Prepared ligands were docked into the active sites of the energy-minimized protein by applying the C-Docker algorithm and adopting the CHARMm force field. The binding energy was computed to assess the enzyme–ligand interactions. The best 10 ligand binding poses were ordered for each ligand according to their C-Docker energies, and the highest ligand binding poses were selected. The root-mean-square deviations (RMSDs) of C-Docker as a docking technique were calculated by superimposing the initial 3D structure and the docked posture of the co-crystallized inhibitor [8].

## 4. Conclusions

The current study’s findings shed light on the variation in the phytochemical profile of *P. racemosa* leaves and stems. LC/MS-based metabolic profiling of the leaf and stem methanol extracts was unveiled for the first time. A total of 61 secondary metabolites were annotated. Extracts showed richness in polyphenolics. In addition, 21 components were identified in leaf and stem essential oils, with eugenol being the most abundant component. The essential oil from *P. racemosa* stems, an agro-industrial waste, exhibited the strongest in vitro *H. pylori* inhibitory activity, which was further validated by in silico molecular modeling of the identified phytoconstituents on *H. pylori* urease. Eugenol, the major active component in leaf and stem essential oils, might be implicated in this action. It could be concluded that *P. racemosa* might offer a natural medicine that exhibits a promising *H. pylori* inhibitory activity and could be accepted by a broad category of customers owing to its natural source. Though, more research is needed to examine additional putative biological actions of *P. racemosa* to discover natural source lead medicines.

## Figures and Tables

**Table 1:** Phytochemical profile of the essential oils of *P. racemosa* leaf (PRL-EO) and stem (PRS-EO).

No.	Rt (min)	Compounds ^a^	Molecular Formula	RI*_exp_* ^b^	RI*_lit_* ^c^	Content %	
						PRL-EO	PRS-EO
1	7.42	α-Thujene	C_10_H_16_	917	917	0.04	-
2	7.61	α-Pinene	C_10_H_16_	938	938	0.89	0.48
3	8.92	β-Pinene	C_10_H_16_	973	973	0.06	-
4	9.05	1-Octen-3-ol (Amyl vinyl carbinol)	C_8_H_16_O	976	976	1.51	0.45
5	9.39	β-Myrcene	C_10_H_16_	990	990	16.30	17.43
6	9.77	α-Phellandrene	C_10_H_16_	1004	1004	1.02	0.58
7	10.16	2-Carene	C_10_H_16_	1015	1014	0.11	0.07
8	10.42	p-Cymene	C_10_H_14_	1023	1023	0.43	0.71
9	10.55	Limonene	C_10_H_16_	1029	1029	7.20	7.70
10	10.63	Eucalyptol	C_10_H_18_O	1033	1033	7.31	5.45
11	11.16	β-Ocimene	C_10_H_16_	1037	1037	0.36	0.92
12	11.50	γ-Terpinene	C_10_H_16_	1060	1060	0.12	0.08
13	12.43	Terpinolene	C_10_H_16_	1090	1090	0.07	0.07
14	15.26	Terpinen-4-ol	C_10_H_18_O	1179	1179	0.34	0.43
15	15.66	α-Terpineol	C_10_H_18_O	1193	1193	1.68	1.49
16	16.05	*n*-Decanal(Capraldehyde)	C_10_H_20_O	1204	1204	0.13	0.24
17	17.61	Chavicol(p-Allylphenol)	C_9_H_10_O	1259	1259	4.18	2.60
18	20.65	Eugenol	C_10_H_12_O_2_	1359	1359	57.84	59.76
19	22.27	Caryophyllene	C_15_H_24_	1424	1424	0.05	0.16
20	24.96	δ-Cadinene	C_15_H_24_	1529	1529	0.05	0.10
21	26.56	Viridiflorol	C_15_H_24_	1591	1591	-	0.10
		Monoterpene hydrocarbons				26.48	28.04
		Oxygenated monoterpenes				9.33	7.37
		Sesquiterpene hydrocarbons				0.1	0.36
		Phenyl propanoids				62.02	62.36
		Others				1.64	0.69
		Total identified %				99.57	98.82

^a^ Compounds are listed based on their elution on an RTX-5MS column. ^b^ RI_exp_, retention indices were determined experimentally on RTX-5MS column relative to a standard hydrocarbon mixture (C8–C28).^c^ RI **_lit_**, published retention indices. Identification of all the compounds was carried out based on a comparison of their mass spectral data (MS) and retention indices (RI) with those of the NIST Mass Spectral Library (2011), Wiley Registry of Mass Spectral Data 8th edition, and the literature.

**Table 3:** Anti-helicobacter pylori activity of essential oils and methanol extracts of *P. racemosa* leaves and stems.

	Inhibition %				
Sample Conc. (µg/mL)	PRL-EO	PRS-EO	PRL-ME	PRS-ME	Clarithromycin
125	100 ± 0	100 ± 0	46.52 ± 0.58	100 ± 0	100 ± 0
62.5	100 ± 0	100 ± 0	19.85 ± 1.8	100 ± 0	100 ± 0
31.25	100 ± 0	100 ± 0	5.74 ± 2.3	100 ± 0	100 ± 0
15.63	83.25 ± 3.1	100 ± 0	0	100 ± 0	100 ± 0
7.81	64.85 ± 1.2	100 ± 0	0	86.32 ±1.5	100 ± 0
3.9	39.17 ± 2.5	100 ± 0	0	55.34 ± 2.4	100 ± 0
1.95	23.14 ± 1.3	92.14 ± 0.95	0	34.38 ± 1.3	100 ± 0
0.98	9.32 ± 1.2	78.95 ± 1.3	0	26.34 ± 0.69	92.45 ± 1.2
0.48	0	56.38 ± 1.6	0	19.3 ± 0.95	87.65 ± 0.58
0.24	0	37.28 ± 2.4	0	7.2 ± 0.83	81.35 ± 1.5
0	0	0	0	0	0
MIC (µg/mL)	31.25	3.9	>125	15.63	1.95

All experiments were carried out in triplicate. Results are expressed as mean ± SD.

**Table 4:** Free binding energies (∆G) calculated in kcal/mol of the identified phytoconstituents within *H. pylori* urease (1E9Y) active site, adopting pH-based and rule-based ionization techniques using Discovery Studio 4.5.

Compound	Free Binding Energy (∆G) (Kcal/mol)	
	pH Based	Rule-Based
Co-crystalized ligand (HAE)	−22.51	−22.51
Decanal	−29.76	−29.76
Eugenol	−29.44	−29.44
α-Terpineol	−23.09	−23.09
δ-Cadinene	−22.68	−22.68
Amyl vinyl	−22.63	−26.57
Chavicol	−21.97	−21.97
Ocimene	−21.91	−21.91
Myrcene	−21.63	−21.63
Terpinolene	−20.24	−20.24
Terpinene	−19.76	−19.76
Phellandrene	−19.71	−19.71
Caryophyllene	−18.83	−18.83
Limonene	−18.71	−18.71
Cymene	−18.51	−18.51
Pinene	−15.48	−15.48
Eucalyptol	−13.31	−13.31

## Data Availability

Data are available upon request from the first author.

## Sample Availability

Samples of the extracts and essential oils are available from the first author.
